# The allometric model in chronic myocardial infarction

**DOI:** 10.1186/1742-4682-9-15

**Published:** 2012-05-11

**Authors:** Maria P Bonomini, Pedro D Arini, Germán E Gonzalez, Bruno Buchholz, Max E Valentinuzzi

**Affiliations:** 1 Instituto de Ingeniería Biomédica (IIBM), Facultad de Ingeniería (FI), Universidad de Buenos Aires (UBA), Buenos Aires, Argentina; 2 Instituto Argentino de Matemática, “Alberto P. Calderón”, CONICET, Buenos Aires, Argentina; 3 Instituto de Fisiopatología Cardiovascular, Facultad de Medicina, Universidad de Buenos Aires (UBA), Buenos Aires, Argentina

**Keywords:** Allometric law, ECG parameters, Chronic infarction extent, Myocardial infarction model

## Abstract

**Background:**

An allometric relationship between different electrocardiogram (ECG) parameters and infarcted ventricular mass was assessed in a myocardial infarction (MI) model in New Zealand rabbits.

**Methods:**

A total of fifteen animals were used, out of which ten underwent left anterior descending coronary artery ligation to induce infarction (7–35% area). Myocardial infarction (MI) evolved and stabilized during a three month-period, after which, rabbits were sacrificed and the injured area was histologically confirmed. Right before sacrifice, ECGs were obtained to correlate several of its parameters to the infarcted mass. The latter was normalized after combining data from planimetry measurements and heart weight. The following ECG parameters were studied: RR and PR intervals, P-wave duration (P_D_), QRS duration (QRS_D_) and amplitude (QRS_A_), Q-wave (Q_A_), R-wave (R_A_) and S-wave (S_A_) amplitudes, T-wave peak amplitude (T_A_), the interval from the peak to the end of the T-wave (T_PE_), ST-segment deviation (ST_A_), QT interval (QT), corrected QT and JT intervals. Corrected QT was analyzed with different correction formulae, i.e., Bazett (QT_B_), Framingham (QT_FRA_), Fridericia (QT_FRI_), Hodge (QT_HO_) and Matsunaga (QT_MA_) and compared thereafter. The former variables and infarcted ventricular mass were then fitted to the allometric equation in terms of deviation from normality, in turn derived after ECGs in 5 healthy rabbits.

**Results:**

Six variables (JT, QT_B_, Q_A_, S_A_, T_A_ and ST_A_) presented statistical differences among leads. QT showed the best allometric fit (*r* = 0.78), followed by T_A_ (*r* = 0.77), ST_A_ (*r* = 0.75), QT_FRA_ (*r* = 0.72), T_PE_ (*r* = 0.69), QT_FRI_ (*r* = 0.68) and QT_MA_ (*r* = 0.68). Corrected QT’s (QT_FRA_, QT_FRI_ and QT_MA_) performed worse than the uncorrected counterpart (QT), the former scaling allometrically with similar goodness of fits.

**Conclusions:**

QT, T_A_, ST_A_ and T_PE_ could possibly be used to assess infarction extent in an old MI event through the allometric model as a first approach. Moreover, the T_PE_ also produced a good allometric scaling, leading to the potential existence of promising allometric indexes to diagnose malignant arrhythmias.

## Background

In a previous paper [1], we briefly gave the basic idea to apply the allometric concept in electrocardiography, that is, and quoting almost verbatim: «Scaling of many biological processes can be described by the allometric equation Y = a*(B_mass_)^b^, where Y is the biological process, B_mass_ the body mass and “a” and “b” are scaling constants. In general, the weights of most individual organs scale as a constant fraction of body mass (i.e., the body mass exponent, “b” equals 1.0). Volume rates, instead (the product of volume and rate), such as cardiac output, ventilation and oxygen uptake, vary with “b” around 0.75. Finally, rates (heart and respiratory rate) scale as “b” close to 0.25. These emergent patterns provide insights into body-size dependent principles that seem to dictate several aspects of design and function across species among all mammals [2,3] » .

Noujaim *et al.*[4] assumed that the heart behaves as a set of “fractal-like” networks tending to minimize propagation time across the conducting system while ensuring a hemodynamically optimal atrioventricular activation sequence. With the mathematical relationship given above and, subsequently, based on previously published values of PR interval, heart rate, and body masses of 541 mammals, they reported as best fit the equation PR = 53 × (B_mass_)^0.24^.

Inspired in the latter report, the following question seems pertinent:

Would a relationship similar to the allometric equation be conceivable, say, between the number of cardiac diseased fibers and any of several ECG parameters when a myocardial infarct (MI) affected the heart?

The objective of this paper tries to find an answer to such question. The Q-wave growth appears as a good candidate because well-known is the fact that normal depolarization suffers with compromised myocardial mass, augmenting the Q-wave and shrinking the R-wave within the QRS complex. Also, additional MI changes include alterations in the T-wave morphology and QRS complex voltages and duration, as well as distortion in intervals such as JT and QT. Many reports confirm this concept, such as Klootwijk, in 1998 [5], Kléber, in 2000 [6], or Balian *et al.*, in 2006 [7], among others. All of these variables and their behaviour on a MI model deserve to be looked at under the allometric light, which seems to maintain interest, especially in general mammalian biology [3,8].

## Methods

### Experimental protocol and procedure to myocardial infarction

An overall of fifteen animals were used in this study. Ten New Zealand rabbits (7 male and 3 female, 1.5–2.5 kg) underwent left anterior descendent (LAD) coronary artery ligation to provoke infarction on a varying extent of myocardial tissue. MI evolved and stabilized during a three month-period after which, rabbits were sacrificed and MI histologically confirmed.

Ventricular infarcted mass in grams was calculated after combining data from planimetric measurements and heart weights. The infarcted mass was then normalized to heart weight in order to compare among different rabbit sizes. Normalized ventricular infarcted mass (VIM_n_) and the ECG parameters mentioned above were, thereafter, fitted to the allometric equation.

This study conformed to the Guide for the Care and Use of Laboratory Animals published by the US National Institutes of Health (NIH Publication No. 85–23, revised 1996).

After anaesthesia with ketamine (75 mg/kg) and Rompun (0,75 mg/kg xylazine) was administered subcutaneously, an endotracheal tube (3 mm inner diameter) was placed to mechanically ventilate the rabbits with room air using a Harvard respirator (25 ml, 34–38 cycles/min). Immediately after, 5% dextrose solution (3 ml/min) was infused through a flexible catheter placed at the marginal vein of one of the ears. Using this venous access throughout the surgical procedure, anaesthesia was maintained by applying additional doses of ketamine and sodium thyopental as needed during the surgical procedure. A left thoracotomy on the fourth intercostal space and a pericardiectomy were performed to expose the heart. Thereafter, a lateral or posterolateral coronary artery branch of the LAD coronary artery was ligated using a curved needle and 6.0 silk thread [9]. The appearance of regional paleness confirmed ventricular wall ischemia. Finally, the thorax was closed with linen thread keeping the order of the different anatomical parts. At the end of the surgical procedure, the animals were placed in a quiet environment for their recovery. Antiobiotic therapy was administered and animals were closely observed during the first 24 hours. Afterwards, animals were placed in individual cages up to the finalization of a three month period.

For the control group, 5 healthy rabbits underwent the above mentioned anaesthetic protocol and ECG recordings were obtained. Analogously to the MI group, the three standard limb leads were recorded.

### Histology and planimetry

To assess infarct size, Masson’s Trichrome (MT) staining was carried out, while infarction was corroborated by Hematoxiline-Eosine (HE). Once excised, hearts were perfused with 10% formaldehyde during 5 min for fixation, remaining in that solution for at least 72 hs. Four millimeter slices were cut out from apex to base and then processed for paraffin embedding and staining. After the latter, fibrotic tissue turned blue, so differentiating itself from healthy tissue, while HE allowed morphological recognition of necrotic tissue.

The stained slices were scanned and planimetric measurements were performed using an image software (Image Pro-Plus 4.5). The area mean percent occupied by the scar tissue at each ventricular level estimated the infarct size [10,11]; these values were expressed as percentage of the total ventricular area while normalized ventricular infarcted mass (VIM_n_) was calculated as follows:

(1) $${\text{VIM}}_{\text{n}}\text{=PIM}\left(\%\right)*\text{VM}\left(\text{gr}\right)/\text{HW}\left(\text{gr}\right)$$

where PIM: planimetric infarcted area (% of the total ventricular area); VM: ventricular mass and HW: heart weight.

### ECG acquisition and preprocessing

Rabbits were heparinized (500 U/Kg IV) 10 minutes before anesthesia by intramuscular injection of ketamine (35 mg/Kg) and lidocaine (5 mg/Kg). All animals were in sinus rhythm at the time of ECG recordings (frontal leads I, II, and III).

ECG data were recorded using instrumentation amplifiers with a gain factor of 1000 and a bandwidth of 0.05–150 Hz. The signals were digitalized at a sampling rate F_s_ = 500 Hz and 12-bit resolution using a digital acquisition board (Lab PC+, National Instruments, Austin, TX, USA). When necessary, a band-stop filter was used to remove 50-Hz. All of the data were acquired and monitored using customized software made in C++.

QRS detection was based on methods described by Hamilton and Tompkins in 1986 [12]. All the ectopic or aberrant beats were automatically rejected by the computer. If needed, baseline corrections were performed by cubic spline interpolation [13].

We computed the running signal average of 30 beats. The template for signal averaging in each ECG channel were created, thereafter, by aligning 30 beats with 98% cross-correlated QRS waveforms using the predefined window and the R-wave peak as the trigger point while, at the same time, correcting for fiducial point jitter. Two rejected beats were the accepted limit on the computation of each final template beat.

### ECG delineation

ECG delineation was accomplished on the template obtained as explained in section “ECG acquisition and preprocessing”. The start and end of the QRS complex were determined by searches on each side of the R-wave for regions where the slope (dV/dt) fell to sufficiently low values. Isoelectric level was taken as the median of all data values preceding the start of the QRS. For the P-wave, we used a modified version of the algorithm described by [14], which is based on finding the peaks and valleys of a 9-point derivative signal. In case of the T-wave delineation, a search was made for the first significant peak of either sign, starting from a point after the end of the QRS. If a suitable peak was found, a straight line was adjusted by least squares from the peak to the tail of the T-wave. The intersection of this line with the isoelectric line was accepted as the T-wave end. ST-segment deviation was defined as the ECG amplitude 50 ms after QRS onset [15].

**Figure 1:**
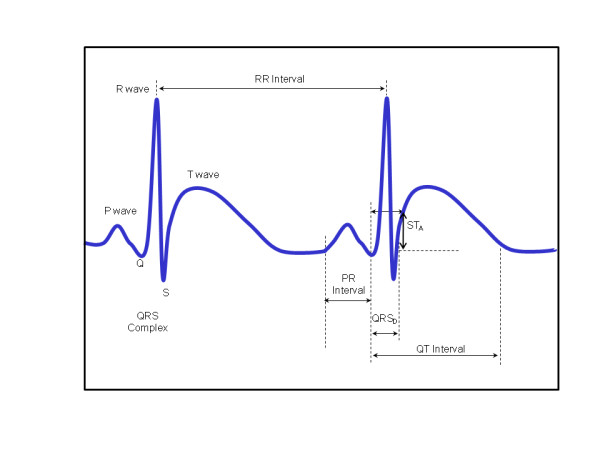
**ECG measurements.** Schematic representation of ECG waves, intervals and segments analyzed in this work.

For all ECG templates, the PR interval, P-wave (P_D_) and QRS duration (QRS_D_), QRS amplitude (QRS_A_), T-wave peak amplitude (T_A_), ST-segment deviation (ST_A_), the time from the peak to the end of the T-wave (T_PE_), QT interval, corrected QT interval (QT_C_) and JT intervals (JT) were calculated. Below the details of the QT corrections are given according to different formulae. All parameters are graphically represented in Figure 1.

### QT_C_ Formulae

The following QT correction formulae were tested:

-Bazett: $QT_{B}=\frac{QT}{\sqrt{RR}}$[16]

-Framingham: $QT_{\mathrm{Fra}}=QT+0.154*(1-RR)$[17]

-Fridericia: $QT_{\mathrm{Fri}}=\frac{QT}{\sqrt[{3}]{QT}}$[18]

-Hodge: $QT_{\mathrm{Ho}}=QT+0.00175*\left(HR-60\right)$[19]

-Matsunaga: $QT_{\mathrm{Ma}}=QT*\frac{\log\left(600\right)}{\log\left(1000*RR\right)}$[20]

where HR = heart rate in bpm, RR = RR interval in s, QT = QT duration in s, QT_C_ = corrected QT interval in s.

### Allometric model – Mathematical setting

Allometry, in general biology, measures the relative growth of a part in relation to the whole living organism. The term was first used by Snell, in 1891 [9], to express the mass of a mammal’s brain as a function of the body mass. The growth velocity of a component *y* is related to the growth velocity of another component (or the whole organism) *x* in a constant way. This was clearly described by von Bertalanffy in 1957 [10]. Thus, the relative rate of change of a given event *y* is proportional to the relative rate of change of body mass or body weight *x*, i.e.,

(2) $$\frac{dy/dt}{y}=B*\frac{dx/dt}{x}$$

After integration and some easy algebraic manipulation, equation (1) becomes

(3) lny=A+B*lnx,

or

(4) $$y=A*x^{B}$$

Originally, *y* was the weight of an organ (heart, stomach, other) and *x* was body weight or mass. The parameters *A* and *B* require numerical estimation by an appropriate procedure usually using empirical information. By the same token, let us hypothesize that the electrocardiographic parameters follow a relationship with the number of ventricular fibers (*N*_*F*_), formally equal to (2), i.e.,

(5) $$ECG_{\mathrm{param}}=a*{\left(N_{F}\right)}^{B}$$

Moreover, in a pathology context, let us relate the “deviation from normality” in terms of ECG with the “deviation from normality” in terms of diseased fibers, modifying the model as in (4),

(6) $$ECG_{D}-ECG_{N}=a*{\left(N_{\mathrm{DF}}\right)}^{\beta }$$

Hence, *y* in equation (2) is replaced by (ECG_*D*_ – ECG_*N*_) in (3), and *N*_*DF*_ in the latter takes the place of *x* in the former. After all, the number of diseased cardiac fibers (ischemic or infarcted or both) are part of the cardiac mass. Besides, since the relationship between diseased fibers and infarcted mass is straightforward, it sounds sensible to state that,

(7) $$N_{\mathrm{DF}}=\gamma *VIM_{n}$$

Hence,

(8) $$ECG_{D}-ECG_{N}{\left(\gamma *VIM_{n}\right)}^{\beta }$$

After taking logarithms of both sides, the latter equation becomes $\log\left(ECG_{D}-ECG_{N}\right)=\left(\log a+\beta *\log\gamma \right)+\beta *\left(\log VIM_{n}\right)\text{,}$ which can be reduced to

(9) $$\log\left(ECG_{D}-ECG_{N}\right)=\delta +\beta *\left(\log VIM_{n}\right)$$

where *δ* = log*α* + β*log*γ*, VIM_n_: log(normalized ventricular infarcted mass) and ECG_*D*_ and ECG_*N*_ are the diseased and healthy counterparts of the following ECG parameters; ST_A_: log(|ST deviation|) in mV; Q_A_: log(|Q-wave amplitude|) in mV; S_A_: log(|S-wave amplitude|) in mV; R_A_: log(|R-wave amplitude|) in mV; QRS_A_: log(|Q| + |R| + |S|) in mV; T_A_: log(T-wave peak amplitude) in mV, T_PE_: log(T-wave time from peak to end); QRS_D_: log(QRS width);.QT: log(QT interval); QTc: log(QT interval corrected by different formulae); RR: log(RR interval); PR: log(PR interval); P_D_: log(P duration). Linear regressions were carried out to obtain numerical values for the intercept *δ* and the slope *β* in the log-log plot.

### Statistical analysis

Data are expressed as mean ± standard deviation (Mean ± SD). To assess statistical significance, leads I, II and III were compared among them. When data did not pass the D’Agostino & Pearson normality test, a non-parametric Kruskall-Wallis test was applied and the Dunn’s post test carried out. Otherwise, one-way ANOVA and Bonferroni post-test were calculated.

## Results

### ECG parameters

ECG parameters were consistent with MI, showing wider and morphologically diverse QRS complexes. QRS voltages suffered in MI, presenting a significant decrease in leads II and III. Due to flattening of the T-wave, ST_A_ resulted very small in Leads II and III, but presented a rounded pattern in Lead I. QRS_D_ was similar in every lead. Neither significant RR nor PR interval changes were seen. P_D_ was longer in lead II, even though no statistical significance was found. Similarly, T_PE_ increased in Lead II but failed to be statistically significant. Lengthening of the JT interval was originated by a flattening of the T-wave, which is much more rounded in the physiological state and offered statistical significance in lead II with respect to the other two leads. The T-wave peak voltage significantly decreased to even become negative in leads II and III, representing the typical T-wave inversion described in chronic infarction [21,22]. Table 1 displays the values for the ECG time-related parameters described above along with the statistical significance values (*p* < 0.05); so does Table 2 for the ECG amplitude-related parameters.

**Table 1:** Electrocardiographic time-related parameters (Mean ± SD) from a 3 month-MI rabbit evolution

	**RR (ms)**	**PR (ms)**	**P**_**D**_**(ms)**	**QRS**_**D**_**(ms)**	**JT (ms)**	**T**_**PE**_**(ms)**
L1	365 ± 92	71 ± 8	29 ± 7	45 ± 6	120 ± 21	59 ± 23
L2	397 ± 99	76 ± 8	32 ± 6	45 ± 4	161 ± 35*	74 ± 25
L3	394 ± 108	72 ± 9	24 ± 6	41 ± 4	127 ± 26	59 ± 24

L1: lead I, L2: lead II, L3: lead III. Statistical significance: *p < 0.05, Lead II against Leads I and III.

**Table 2:** Electrocardiographic amplitude-related parameters (Mean ± SD) from a 3 month-MI rabbit evolution

	**Q**_**A**_**(mV)**	**R**_**A**_**(mV)**	**S**_**A**_**(mV)**	**QRS**_**A**_**(mV)**	**ST**_**A**_**(mV)**	**T**_**A**_**(mV)**
L1	−0.50 ± 0.33*	0.21 ± 0.23	−0.12 ± 0.36*	0.83 ± 0.92	0.08 ± 0.04*	0.10 ± 0.12*
L2	−0.29 ± 0.23	0.22 ± 0.16	0.01 ± 0.05	0.52 ± 0.44	0.01 ± 0.05	−0.03 ± 0.09
L3	−0.12 ± 0.15	0.37 ± 0.24	−0.02 ± 0.03	0.51 ± 0.42	−0.02 ± 0.03	−0.05 ± 0.07

L1: lead I, L2: lead II, L3: lead III. Statistical significance: *p < 0.05, Lead I against Lead III.

QT interval did not show significant differences among leads. On the other hand, among all the correction formulae tested (Bazett, Framingham, Friderica, Hodges and Matsunaga), the only one that led to statistical significance was Bazett’s formula, with a clear differentiation from Leads II and III. Table 3 summarizes the QT and QT corrected values for every lead.

**Table 3:** Corrected and uncorrected QT intervals (Mean ± SD) from a 3 month-MI rabbit evolution

	**QT (ms)**	**QT**_**B**_**(ms)**	**QT**_**FRA**_**(ms)**	**QT**_**FRI**_**(ms)**	**QT**_**HO**_**(ms)**	**QT**_**MA**_**(ms)**
L1	182 ± 24	296 ± 25	272 ± 16	242 ± 23	359 ± 50	189 ± 20
L2	187 ± 35	293 ± 42	265 ± 19	235 ± 28	350 ± 54	184 ± 25
L3	169 ± 29	272 ± 28*^2^	262 ± 20	231 ± 25	347 ± 57	182 ± 25

L1: lead I, L2: lead II, L3: lead III. QT_FRA_: Framingham correction, QT_FRI_: Fridericia correction, QT_MA_: Matsunaga correction, QT_HO_: Hodge correction. Statistical significance: *p < 0.05, Lead III versus Lead II.

### Allometric fits

**Figure 2:**
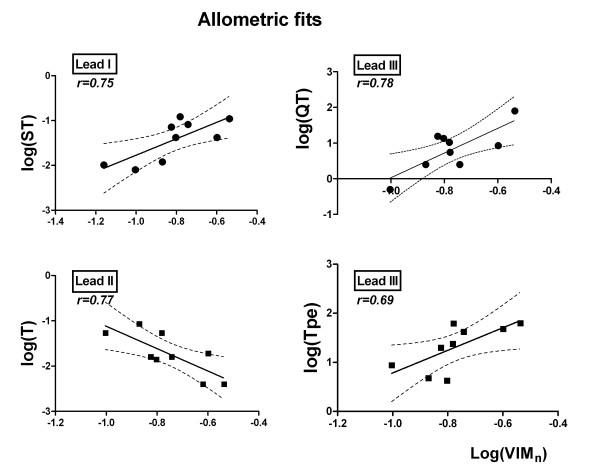
**Allometric scaling of ECG parameters.** Allometric scaling of ST_A_, T_A_, QT and T_PE_ versus infarction extent. Notice the negative slope of T_A_ with infarction extent.

In general terms, 7 out of 18 measures presented a clear allometric scaling with VIM_n_. Lead I contained 4 out of 7 such parameters (ST_A_, QT_FRA_, QT_FRI_ and QT_MA_), followed by Lead III with 2 (QT and T_PE_) and Lead II (T_A_) with only one. This lead preference for each parameter explains the way data are presented, on a lead separated-basis. The variable that best fitted the allometric equation was QT (*r* = 0.78) followed by T_A_ (*r* = 0.77), ST_A_ (*r* = 0.75), QT_FRA_ (r = 0.72), T_PE_ (*r* = 0.69), QT_FRI_ (*r* = 0.68) and QT_MA_ (*r* = 0.68), all of these with statistical significance (p < 0.05). It is worth noting that T_A_ presented a negative allometric fit with VIM_n_ (*β* = −2.474 ± 0.7736), meaning that the value of T-voltage decreased as VIM_n_ increased. A similar fit was found for the Q-wave amplitude (*β* = −0.6352 ± 0.2849), even though it failed to account for statistical significance (p = 0.0610). On the contrary, S_A_ increased along with VIM_n_ producing a positive slope (*β =* 1.202 ± 0.6668) in the allometric equation. This opposite trend would explain the change in morphology of QRS complexes, shifting from a QR to an RS pattern. ST_A_ deviation in Lead I displayed a good linear regression with VIM_n_ in a log-log representation. The slope for this regression turned up to be positive, leading to an ST_A_ increase with infarct size. On the other hand, T_PE_ in Lead III also increased with VIM_n_ reflecting important changes in T-wave morphology, mostly due to the flattening of the T-wave, which is normally rounded in rabbits. Figure 2 displays the allometric scaling of the mentioned variables QT, T_A_, T_PE_ and ST_A_. Adjustments are shown as well as 95% confidence limits (broken lines).

**Figure 3:**
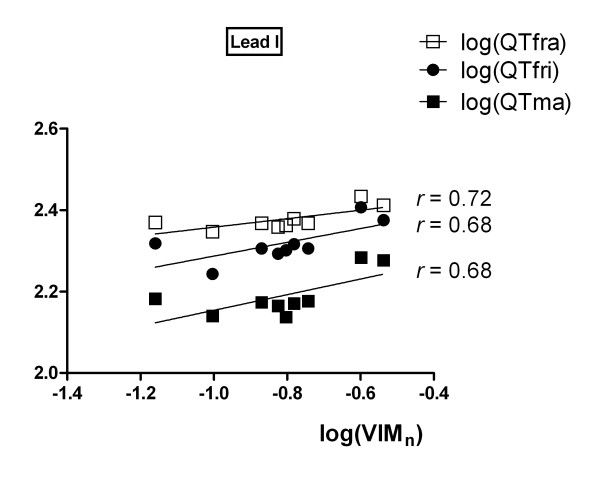
**Allometric scaling of QT and QTc parameters.** Corrected QT allometric fits. QTfra: Framingham correction formula, QTfri: Fridericia correction formula, QTma: Matsunaga correction formula.

QT corrections presented allometric fits with VIM_n_ as well, and were analyzed in a different graph (Figure 3) for clarity sake. In lead I, the Framingham, Matsunaga and Fridericia formulae corrections for heart rate allometrically scaled to VIM_n_. Accordingly, all the corrected QT intervals displayed a slope smaller than one, near 0.25, as cited in the bibliography [2-4], being the uncorrected QT the only one that deviated from this pattern (*β =* 3.464 ± 1.072).

The remaining variables, RR, PR, P_D_, QRS_D_, JT, QT_HO_, QT_B_, QRS_A_ and Q_A_, R_A_ and S_A_ did not present a fit with a good statistical significance (p > 0.05). Moreover, they kept almost constant for all MI extents (data not shown).

A complete description of the allometric fits can be found in Table 4, where all parameters together with the slope *β*, the origin intercept *δ,* the goodness of fit *r* and the statistical significance *p-*value are shown. Each parameter was chosen from the lead at which the best allometric fit was produced.

**Table 4:** Linear regressions of all ECG parameters for a 3 month-MI rabbit evolution grouped by leads

**Lead**	**Parameter**	***β (Mean ± SD)***	***δ (Mean ± SD)***	***r***	***p-value***
**L3**	QT	3.464 ± 1.072	3.494 ± 0.8387	0.78	0.0145
**L2**	T_A_	−2.474 ± 0.7736	−3.593 ± 0.5926	0.77	0.0151
**L1**	ST_A_	1.832 ± 0.5997	0.06115 ± 0.4994	0.75	0.0185
**L1**	QT_FRA_	0.1042 ± 0.03840	2.462 ± 0.03198	0.72	0.0300
**L3**	T_PE_	2.303 ± 0.9132	3.087 ± 0.7142	0.69	0.0397
**L1**	QT_FRI_	0.1691 ± 0.06926	2.456 ± 0.05769	0.68	0.0447
**L1**	QT_MA_	0.1918 ± 0.07858	2.346 ± 0.06545	0.68	0.0447
**L1**	QT_B_	0.1384 ± 0.0617	2.531 ± 0.05142	0.64	0.0600
**L3**	Q_A_	−0.6352 ± 0.2849	−0.8878 ± 0.2228	0.64	0.0610
**L3**	S_A_	1.202 ± 0.6668	0.03635 ± 0.5215	0.55	0.1145
**L2**	P_D_	1.011 ± 0.5893	1.510 ± 0.4514	0.54	0.1299
**L1**	QRS_A_	1.210 ± 0.7190	0.2376 ± 0.5988	0.53	0.1362
**L3**	QRS_D_	0.5699 ± 0.3995	1.566 ± 0.3124	0.47	0.1968
**L1**	RR	0.5025 ± 0.3669	2.675 ± 0.3056	0.45	0.2131
**L2**	PR	−0.7553 ± 0.7338	0.3425 ± 0.5621	0.36	0.3376
**L1**	R_A_	0.7621 ± 0.7972	0.09869 ± 0.6639	0.34	0.3709
**L3**	JT	0.5296 ± 0.6854	1.821 ± 0.5360	0.26	0.4650
**L3**	QT_HO_	0.1448 ± 0.3730	2.350 ± 0.2917	0.14	0.7095

*β*: slope of linear regression, *δ*: Y-intercept when X = 0 of linear regression, *r*: correlation coefficient, *p-value:* statistical significance of linear fit. L1: Lead I, L2: Lead II, L3: Lead III, QT_FRA_: Framingham correction formula, QT_MA_: Matsunaga correction formula, QT_B_: Bazett correction formula, QT_FRI_: Fridericia correction formula, QT_HO_: Hodges correction formula, QT: QT interval, T_A_: amplitude of T-wave, ST_A_: amplitude of ST segment deviation, T_PE_: time from peak to end of the T-wave, Q_A_: amplitude of Q-wave, S_A_: amplitude of S-wave, R_A_: amplitude of R-wave, QRS_A_: amplitude of QRS complex, QRS_D_: duration of QRS complex, RR: RR interval, PR: PR interval, JT: JT interval.

## Discussion

### Findings of the present study

An allometric study was presented here in order to analyze the allometric scaling of different ECG parameters with respect to the infarction extent in a chronic experimental MI rabbit model. The idea behind this work was to find ECG data to estimate myocardial injured area as a first, simple and inexpensive step before resorting to more complex or invasive technologies, such as magnetic resonance imaging or radioscopic procedures. The chief findings of this study were as follows:

1- Six variables (JT, QT_B_, Q_A_, S_A_, T_A_ and ST_A_) showed statistical differences among leads. Lead I versus Lead III contained 5 out of 6 of these statistical differences (QT_B_, Q_A_, S_A_, T_A_ and ST_A_), while JT presented differences in Lead II with respect to Lead I. Most of these variables turned out to be amplitude-related (Q_A_, S_A_, T_A_ and ST_A_) and, probably, the lead with the most evident changes was related to infarct localization.

2- Seven out of eighteen parameters presented a significant allometric fit (p < 0.05). Lead I accounted for the majority of them (ST_A_, QT_FRA_, QT_FRI_, QT_MA_), followed by Lead III (QT, T_PE_) and Lead II (T_A_). See Table 4, where it can also be seen that QT_B_ and Q_A_ showed borderline significances and still with a good fit.

3- The variables that best adjusted to the allometric equation were QT, T_A_ and S_TA_ with a goodness of fit enough to assess infarction size within a sensitive range *(r* = 0.78*, r* = 0.77*, r* = 0.75, respectively.

### Significance and interpretation of the data

Ventricular changes found during the chronic MI state can be split into ventricular *depolarization* and *repolarization* changes.

#### Ventricular Depolarization Changes

In chronic MI hearts, QRS morphologies changed [21,22]. Even though not statistical significant, a negative Q-voltage slope and a positive S-voltage slope in the allometric equation suggest a shift from the QR to the RS morphology.

Surprisingly, QRS_D_ did not fit well the allometric equation, as it would have been expected. We cannot find a good explanation for such behaviour. Evidence refers to the prognostic importance of QRS duration in acute and chronic myocardial infarction [23,24]; hence, further investigation would be needed.

#### Ventricular Repolarization Changes

The T-wave showed a change in morphology, too, becoming flat instead of rounded. Moreover, negative T-wave morphologies appeared, mostly in lead II and III. These changes forced the JT interval to be longer and T_PE_ to be longer as well as a smaller T_A_. On the other hand, QT interval and T_PE_ presented a good allometric scaling. It is tempting to link this observation with the fact that increased arrhythmogenesis is related to MI hearts, as reported elsewhere [25-32].

The JT-interval could be considered as a total ventricular repolarization process. In our study, such interval was increased in MI, which might indicate a differential lengthening or shortening of the action potential duration (APD) in some myocardial areas, so reflecting the range of times at which action potentials end and, therefore, it would be an expression of APD heterogeneity [33]. However, JT showed a bad allometric scaling and, at least based on this study, we could not establish the allometric behaviour of this parameter.

Uncorrected QT offered the best fit, in agreement with the literature on QT and ischaemia. With transmural ischaemia, the T-wave axis shifts in the opposite direction towards the region of epicardial involvement, being often a sign of infarction. Such T-waves are tall and peaked, frequently with long QT intervals [34]. These changes may assist in the localization of ischaemia and also in the quantification of the injury size. It is worth noting that the slope of all corrected QT where about 0.25, similar to those found in the literature related to allometry; the uncorrected QT was the only one out of this pattern (*β =* 3.464 ± 1.072) [2,4].

Surprisingly, ST_A_ deviation in Lead I displayed a good linear regression with VIM_n_ in a log-log representation. This fact was opposite to our expectations since in humans, ST deviation tends to normalize in chronic MI. Nevertheless, ST-segment in rodents (especially in rats, and less markedly in rabbits) shows a different behaviour in the physiological case [35]. Thus it is plausible to think that these differences will still hold for the pathological cases.

In addition, someone may think that a multilead approach would be a good methodology to measure the parameters. However, in our case, the number of leads was only three and we think that is not enough number to guarantee that kind of analysis. On top of that, we did not have a criterion to select one or the other lead.

Finally, it should be underlined that the reference set of normal data, even though they appear at first sight as a good idea (see equation #4), did not significantly changed the results when using a direct relationship such as

(10) $$ECG_{\mathrm{param}}=a*{\left(N_{\mathrm{DF}}\right)}^{\beta }$$

from which numerical results are not shown in this report (see also above, Findings of the present study, item 3).

### Study implications and future work

The first half of the T-wave is mainly related to epicardial APD, whereas the second half is to the endocardial and mid-myocardial APD [36,37]. As a result, at the peak of the T-wave the transmural voltage gradient reaches the maximum and, therefore, the descending limb represents the extent of transmural dispersion of ventricular repolarization. Therefore, great is the importance of T_PE_ producing a good allometric scaling, leading to a new and attractive future work, which is the search of allometric indexes for diagnosing malignant arrhythmias.

QT, T_A_ and ST_A_ showed the strongest allometric behavior. This implies that chronic MI extents could be roughly assessed by feeding the allometric equation with any of these parameters. Whether one outstands from the others or not and hence, whether the allometric equation should be fed with any of these parameters individually or collectively with any combination of them, remains open.

Another consideration is pertinent regarding MI localization. Data presented in this work is valid for anteroseptal injury only. Thus, the search of electrocardiographic allometric scaling on different MI localizations would complete this study.

### Study limitations

In the analysis of several electrocardiographic parameters, most of the ECG time-related and amplitude-related measurements have been taken into account. Nevertheless, other approaches for QT correction should be explored, like the individual correction suggested by Malik [38] or the correction for animals carried out by Valentinuzzi [39]. On the other hand, the relations found here are strictly related to infarcts originated from LAD coronary artery ligation, not covering other MI localizations. Besides, a wider range of infarction extent would be desirable, allometrically speaking, but not possible due to the extremely low survival rates at MI extents greater than 40%.

## Conclusions

The objectives of this paper were met, since ECG parameters related to depolarization and repolarization phases showed a certain degree of adjustment to the allometric equation, some of them rather good. The parameters that best allometrically scaled were QT, T_A_ and ST_A_, with a goodness of fit enough to assess infarction size within an acceptable range. Besides, the T_PE_ produced a good allometric scaling, leading to the potential existence of promising indexes to diagnose malignant arrhythmias.

## Competing interests

The authors declare that they have no competing interests.

## Authors’ contributions

MPB carried out the animal experiments and drafted the manuscript. MPB, PDA and MEV made key contributions to the conception and design, analysis and interpretation of data, and drafting of the manuscript. GEG and BB run the reference series. All authors read and approved the final manuscript.
